# Boric Acid Induces Oxidative Damage and Apoptosis Through SEMA3A/PLXNA1/NRP1 Signalling Pathway in U251 Glioblastoma Cell

**DOI:** 10.1111/jcmm.70578

**Published:** 2025-05-03

**Authors:** Ezgi Kar, Zeynep Övenler, Ceyhan Hacıoğlu, Fatih Kar

**Affiliations:** ^1^ Department of Nutrition and Dietetics, Faculty of Health Sciences Kutahya Health Sciences University Kutahya Turkey; ^2^ Faculty of Medicine Kutahya Health Sciences University Kutahya Turkey; ^3^ Department of Medical Biochemistry, Faculty of Medicine Duzce University Duzce Turkey; ^4^ Department of Medical Biochemistry, Faculty of Medicine Kutahya Health Sciences University Kutahya Turkey

**Keywords:** apoptosis, boric acid, glioblastoma, oxidative damage, semaphorin

## Abstract

Glioblastoma is one of the deadliest cancers with a very low chance of survival. Glioblastomas have a poor prognosis because of their infiltrative nature, which makes them difficult to totally isolate with rigorous surgery, radiation, and chemotherapy. Our aim in this study was to investigate the efficacy of boric acid, which has anti‐cancer properties, on glioblastoma, which has very limited treatment options. U251 human glioblastoma cell lines were treated with IC25 (15.62 μg/mL), IC50 (31.25 μg/mL) and IC75 (62.5 μg/mL) doses of boric acid. Cell viability and proliferation levels were tested. At the same time, the activity of boric acid on cells was tested through oxidative stress, apoptosis, and semaphorin signalling pathway parameters. Our findings indicate that boric acid induced dose‐dependent oxidative stress, cellular growth inhibition, apoptosis and morphological changes in U251 cells. Additionally, treatments with increasing amounts of boric acid resulted in a rise in the production of biomarkers of the semaphorin pathway, which may limit cell growth and proliferation. We found that boric acid activates apoptosis by triggering ROS formation at high doses and at the same time inhibits cell proliferation by increasing semaphorin signalling pathway expressions. Boric acid may act as an anti‐cancer agent by activating different mechanisms in a dose‐dependent manner.

## 1 Introduction

Glioblastoma multiforme (GBM) is a primary malignant brain tumour that arises within intracranial tissues or glial cells, and these cells play a vital role in providing neurons with essential nutrients and oxygen [1]. GBM leads to premature mortality in adults under the age of 60 and children at 15 years old, with a higher mortality rate. The median survival time is approximately 12.1 to 14.6 months, and only 2%–3% of GBM patients survive up to two years following aggressive tumour resection (surgery), radiation therapy or chemotherapy [2]. The infiltrative nature of glioma cells is the underlying reason for the unfavourable prognosis of these malignancies. This not only leads to a dismal prognosis but also has a direct impact on the neurological function of the brain, psychological well‐being, and overall quality of life, causing significant consequential challenges in GBM patients [3, 4]. A more comprehensive understanding is needed regarding the epidemiology, physicochemical attributes of drugs, microenvironment, and resistance characteristics of glioma cells, and innovative drug carrier systems, along with their delivery methods. Given the challenges experienced in drug response over the past few decades within pharmaceutical clinical research, there is a clear and urgent requirement for new targeted therapies.

Semaphorins (SEMA) constitute an extensive family of glycoproteins, categorised into eight classes, with members that are either transmembrane, secreted or glycosylphosphatidylinositol‐linked to the membrane [5]. Originally recognised for their role in guiding axonal growth and navigation, SEMAs play a crucial part in the progress of neuronal circuits [6]. The majority of SEMA signals are transmitted through interactions with receptors from the plexin (PLXN) family [7]. These receptors can bind to SEMAs independently or in conjunction with co‐receptors, such as neuropilins (NRPs). SEMA signals extend outside axon guidance, influencing a diverse array of functions. These can be found in both healthy and pathological settings, and they include angiogenesis, cell migration, substrate adherence, cell viability, cell growth and immunological response [6]. The initial indication of SEMAs' role in tumorigenesis emerged with the identification of SEMA3A and SEMA3F genes in the 3p21 chromosomal region. A significant discovery was the detection of a deletion in this region in the majority of small‐cell lung cancers [8, 9]. Over the past decade, a growing frame of experimental evidence has highlighted the significant role of SEMAs in various types of cancer, comprising GBM [10, 11, 12]. The initial indication of SEMAs and their receptors playing a role in GBM was founded on their expression in human glioma cells [13].

Reactive oxygen species (ROS) play a critical role in modulating cellular constancy by inducing diverse signalling pathways. Under normal circumstances, redox systems work to avert oxidative damage to cells. Nevertheless, gliomagenesis severely impairs cellular oxidation–reduction mechanisms [14]. The generation of ROS in tumour cells not only impacts the cell cycle, leading to tumour progression and drug resistance in GBM, but also induces cell death programmes like apoptosis and autophagy when produced excessively. Given the heightened metabolic rate and increased ROS levels in GBM cells, their metabolic adaptation becomes crucial in resisting oxidative stress‐induced cell death [15]. Therefore, optimal therapy for gliomas and other types of cancer may be systems that both modulate oxidative stress and trigger programmed cell death mechanisms, and it is clear that their detailed investigation is warranted.

Boron (B), having an atomic number of five, is situated in the 3A group of the periodic table, where it stands as the sole non‐metal element. It occurs naturally in compounds termed oxygen‐bound borates, with the most prevalent ones including boron oxide, salts of boric acid (such as sodium tetraborates, commonly known as borax), and boric acid (BA) itself [16]. In the human body, inorganic borate compounds, primarily as boric acid, are present [17] and almost the entire amount of ingested BA from the diet is absorbed through the gastrointestinal tract. Absorption of BA through the gastrointestinal tract from the diet approaches close to 100% [18]. Experimental studies have tested the applications of BA, revealing significant improvements in both humans and animals [19, 20, 21, 22]. After all these biological activities and non‐toxic properties of BA have been revealed, studies on its anti‐cancer effect have also increased. BA has garnered increased interest among cancer researchers, particularly following the discovery of boron neutron capture therapy, a therapeutic technique for treating invasive malignant tumours using a radiation source and boron [23]. Additionally, the development of bortezomib, a boron‐containing antineoplastic drug, has contributed to this heightened interest [24, 25, 26]. Considering the cancer studies conducted with BA in the literature, it is seen that its efficacy on different cancer types such as liver, lung, prostate, breast, ovarian, colon and skin cancer has been examined [27, 28, 29, 30]. A recent study investigated the anti‐cancer effects of BA in acute lymphocytic leukaemia cells [31].

In the present study, we investigated the anti‐tumorigenic effects of BA through the semaphorin/plexin/neurophilin pathway, oxidative stress and apoptosis, which have been found to control GBM progression in U251 cells.

## 2 Materials and Methods

### 2.1 Cell Culture

U251 human malignant glioblastoma cell culture lines, purchased from the American Type Culture Collection (ATCC, USA), were cultured in Dulbecco's Modified Eagle Medium (DMEM) (Sigma‐Aldrich, GmbH, Germany) supplemented with 10% Fetal Bovine Serum (FBS; Gibco, Grand Island, NY) and 1% (v/v) antibiotic‐antimycotic (penicillin–streptomycin) solution (A5955, Sigma‐Aldrich). The cells were grown at 37°C in a humidified incubator with 5% CO_2_. Initially, the cells were plated in 25 cm^2^ cell culture flasks and transported to 75 cm^2^ flasks when they reached the appropriate size. Passaging was carried out by rinsing with Phosphate Buffer Saline (PBS) and using 0.25% trypsin and ethylenediaminetetraacetic acid (EDTA) (T4049, Sigma‐Aldrich).

### 2.2 Cell Viability and Proliferation Analyses

50 mM BA solution was freshly prepared on the day of the experiment as a stock solution and diluted and passed through 0.21 μm filters. Cells were seeded in 96‐well plates with 5 × 10^3^ cells in each well and incubated overnight. The cells were then treated with BA concentrations of 12.5, 25, 50, 100, 200, 400 and 800 μg/mL for incubation periods of 24, 48, and 72 h. With the expiration of the incubation time, a cell viability test was performed with 3‐(4,5 dimethylthiazol‐2‐Yl)‐2,5‐diphenyltetrazolium bromide (MTT).

The following formula was used to calculate the cell viability percentages:

$$\left(\text{Absorbance of}\;\mathrm{BA}\;\text{added cells}-\text{Blind absorbance}\right)/\left(\text{Absorbance of control cells}-\text{Blind absorbance}\right)\times 100.$$

With the MTT results, three different BA concentrations (IC25, IC50 and IC75) were determined as 15.62, 31.25 and 62.5 μg/mL, and the experimental procedure was continued over these concentrations. The incubation period was determined as 24 h.

### 2.3 Cell Morphology Assay

To observe the effects of BA on U251 cells, image analyses of the cells were obtained with an inverted microscope. Cells were grown in a 6‐well plate at 2 × 10^5^ cells per well, then treated with three different BA concentrations for 24 h. After the fixation and washing processes, an inverted microscope (Olympus microscope and DP80 digital camera) was used to take pictures of the cells.

### 2.4 Cell Lysate Preparing Process and Quantification of Protein Levels

Cell lysates of BA‐treated cells were arranged for biochemical analysis. Cells (5 × 10^3^ cells/well) were scattered for one night in a 12‐well plate and then preserved with BA concentrations (15.62, 31.25 and 62.5 μg/mL) for 24 h, except for the control group. After the incubation time, the cells were cleaned with phosphate buffer (PBS, pH 7 at 4°C) and centrifuged at 500× *g* for 10 min at 4°C in a refrigerated centrifuge (The Sigma 1‐14K centrifuge, Sigma Laborzentrifugen GmbH, Germany) after the adherent cells were detached with trypsin. The cells were then collected into 1.5 mL Eppendorf tubes. The supernatant was waste, and the pellet was collected. The pellet was then rinsed with PBS again and diluted with 100 mL radioimmunoprecipitation assay buffer (RIPA, R0278, Sigma‐Aldrich) with protease inhibitor cocktail (P8340, Sigma‐Aldrich). Cell residues in the samples were removed by centrifugation at 10,000× *g* for 45 min at 4°C. Protein levels in control and BA‐treated cell lysates were measured in accordance with the Lowry method [32].

### 2.5 Biochemical Measurements

Biochemical measurements were performed in cell lysates to evaluate oxidative and apoptotic mechanisms in U251 cells treated with different doses of BA and to see the effects of SEMA/PLXN/NRP signalling pathway.

Total antioxidant level (TAS, RL0017) and total oxidant level (TOS, RL0024) were assessed spectrophotometrically using commercial kits obtained from Rel Assay Diagnostics kits (Gaziantep, Türkiye) to assess oxidative damage balance. The measurements were carried out using a spectrophotometer (S1010/S1020 Spectrophotometer, Techcomp, China). Reduced glutathione (GSH, BT Lab, Code: EA0142Hu, China) levels were also evaluated by Enzyme‐Linked ImmunoSorbent Assay (ELISA) with commercial kits' protocols. In addition, Oxidative Stress Index (OSI) values were calculated by proportioning TOS levels to TAS levels.

Caspase‐3 (CASP3, BT Lab, Code: E4804Hu, China), cytochrome‐c (CYCS, Cloud‐Clone Corp., Code: SEA594Mi, China), BAX (BT Lab, Code: E4977Hu, China) and Bcl‐2 (BT Lab, Code: E4102hu, China) levels were measured to evaluate apoptosis processes with commercial ELISA kits' protocols.

SEMA3A (Cloud‐Clone Corp., Code: SEL917Hu, China), PXNA1 (BT Lab, Code: E2631Hu, China) and NRP1 (BT Lab, Code: E2101Hu, China) levels were also measured by the ELISA method. The measurements were carried out using an ELISA plate reader (Multiskan SkyHigh Microplate Spectrophotometer, Thermo Scientific, USA).

### 2.6 Molecular Analyses

The SEMA/PLXN/NRP signalling pathway was also evaluated by gene analyses. RNA was isolated from the samples, and Real Time Polymerase Chain Reaction (qRT‐PCR) was performed. For qRT‐PCR measurements, the isolation of RNA from cell lysates was initially performed, adhering to the relevant commercial kit protocol (A.B.T. Blood/Tissue RNA Purification Kit, A.B.T. Biotech, Türkiye). The extracted RNAs were transformed into complementary DNA (cDNA) within the PCR device (SimpliAmp Thermal Cycler, Thermo Fisher Scientific, USA) using the relevant commercial kit protocol (A.B.T. cDNA Synthesis Kit with RNase Inh. (High Capacity), A.B.T. Biotech, Türkiye). This step was taken to stabilise the RNAs before conducting qRT‐PCR measurements. (qRT‐PCR) measurements were conducted following the protocol of A.B.T 2X qPCR SYBR‐Green Master Mix (A.B.T. Biotech, Türkiye). Primer sequences specific to each gene were used (Table 1), and Ct values were calculated to determine the expression of each gene. Analyses were performed with at least three replicates. The β‐actin gene served as a control for calculations, and relative quantities were determined using the formula 2^−ΔΔCT^.

**TABLE 1:** Primer sequences of qRT‐PCR measurements.

	Primer
β‐Actin forward	TGTTTGAGACCTTCAACACCC
β‐Actin reverse	AGCACTGTGTTGGCGTACAG
SEMA3A forward	GGCGAGACTTTGCTATCTTC
SEMA3A reverse	GCTATACATACACACGGCTG
PLXNA1 forward	ACTACCGGACATATGCCATGC
PLXNA1 reverse	CTCCTTGGCCTGGGTGACCG
NRP1 forward	ATGGAGAGGGGGCTGCCG
NRP1 reverse	CAACATCAGGGAATCCATCCC

### 2.7 Statistical Analysis

The entire data set obtained as a result of the analyses was first subjected to the Shapiro–Wilk normality test. Data with a normal distribution were assessed using one‐way analysis of variance (ANOVA) and subsequently analysed using the Tukey post hoc test for multiple comparisons. A significance level of *p* < 0.05 was deemed statistically significant. Statistical analysis was conducted using SPSS Version 21.0 (IBM Corporation, Armonk, New York, USA) and GraphPad Prism 7 software (GraphPad Software Inc., San Diego, CA, USA). Results were expressed as mean ± standard deviation (SD).

## 3 Results

### 3.1 Cell Viability and Morphology Results

**FIGURE 1:**
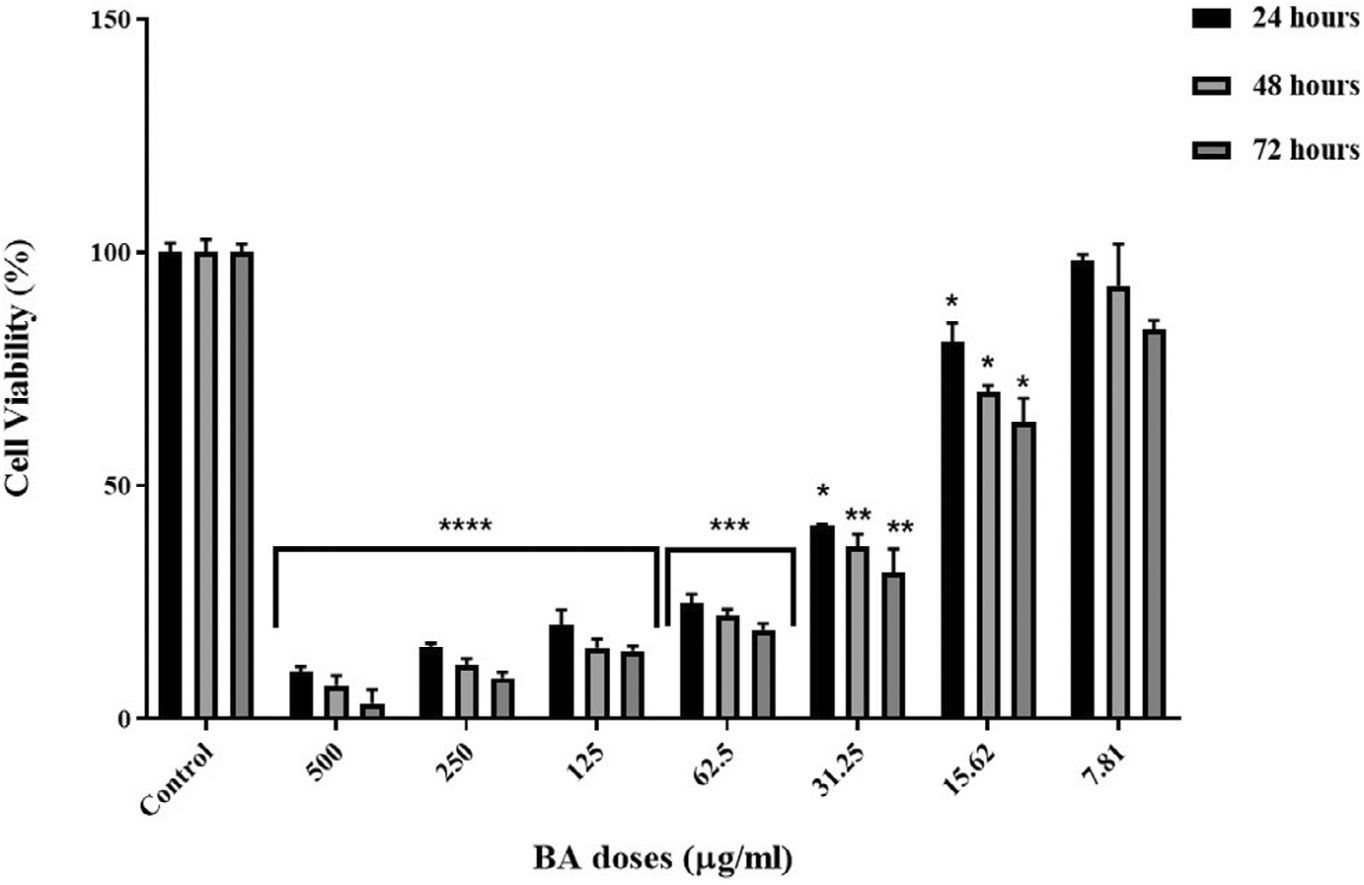
MTT viability analysis results of U251 cells. *p < 0.05, **p < 0.01, ***p < 0.001 and ****p < 0.0001 compared to the control group. The results are presented as the mean ± SD.

**FIGURE 2:**
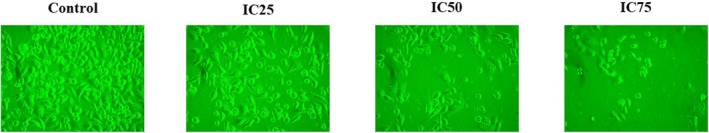
Effects of BA on cell morphology in U251 cells. IC25 15.62, IC50 31.25, and IC75 62.5 μg/mL, respectively.

For the activity of BA on cell viability, 3 doses, IC25, IC50, and IC75, were tested. These were determined as 15.62, 31.25, and 62.5 μg/mL, respectively (Figure 1). Using an inverted microscope, it was determined that the viability and number of cells decreased in a dose‐dependent manner (Figure 2). The period of dramatic decrease in cell viability was determined as 24 h.

### 3.2 Biochemical Measurements Results

**FIGURE 3:**
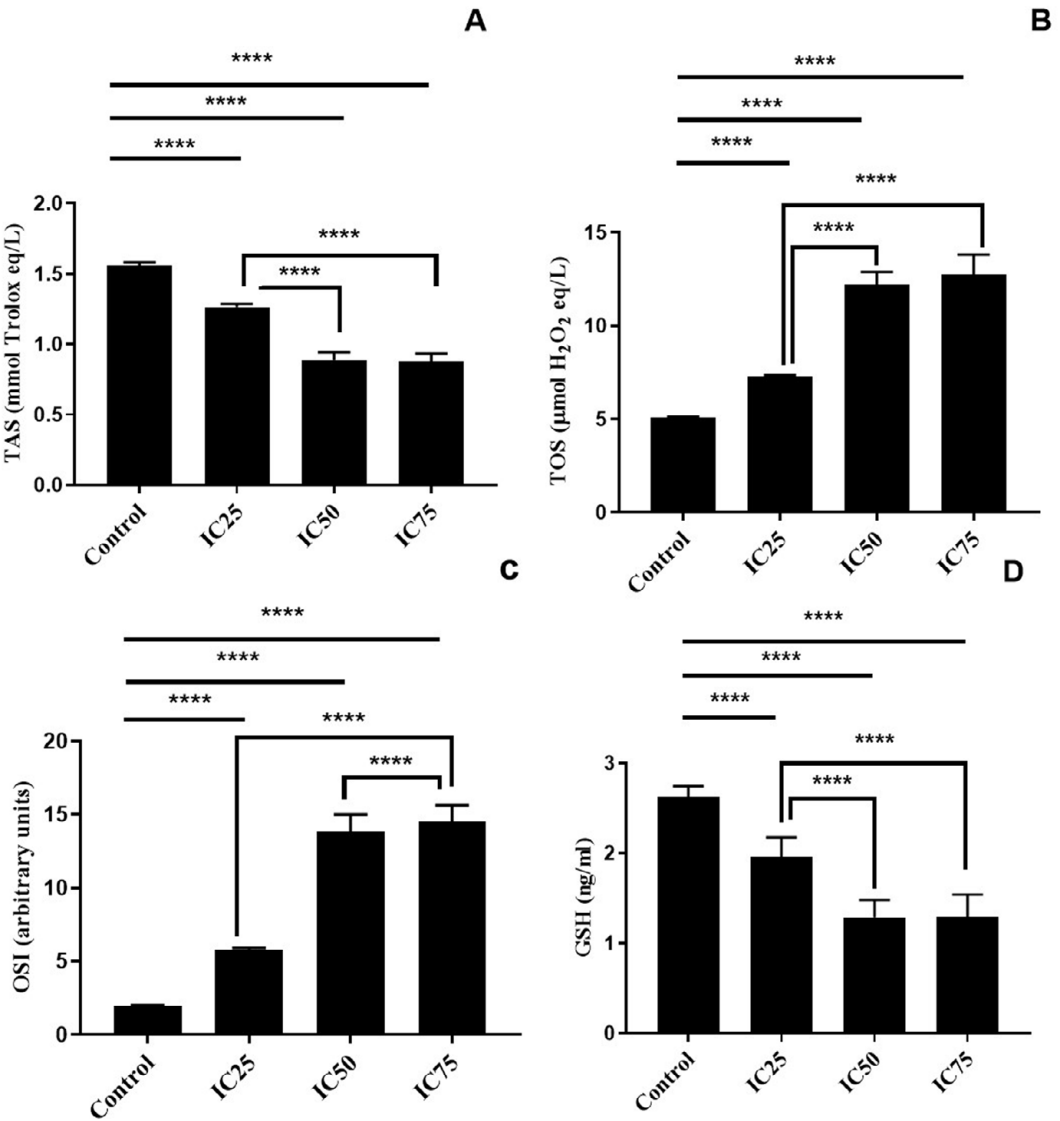
Oxidative damage panel of U251 cells with BA treatment. ****p < 0.0001, IC25 15.62, IC50 31.25, and IC75 62.5 μg/mL BA, respectively. The results are presented as the mean ± SD.

Oxidative stress parameters of the cells were evaluated in a BA dose‐dependent manner. It was determined that the antioxidant balance of the cells deteriorated and TAS values decreased as the BA dose increased (Figure 3A). Compared to the control group, TAS levels were significantly decreased at all IC25, IC50 and IC75 doses (*p* < 0.0001, Figure 3A). There was a statistically significant decrease in TAS levels at IC50 and IC75 doses compared to the IC25 dose (*p* < 0.0001, Figure 3A). In parallel with these results, TOS and OSI levels were significantly increased in BA‐treated cells compared to control cells as a result of the deterioration of the antioxidant balance in the oxidant direction (*p* < 0.0001, Figure 3B,C, respectively). At the same time, the statistically significant increase in IC50 and IC75 treated cells compared to IC25 treated cells supports the dose‐dependent effect (*p* < 0.0001, Figure 3B,C, respectively). GSH levels, an important defence and antioxidant molecule of cells, were significantly decreased in BA‐treated cells compared to control cells (*p* < 0.0001, Figure 3D). In cells treated with IC50 and IC75, a more significant decrease occurred compared to IC25.

**FIGURE 4:**
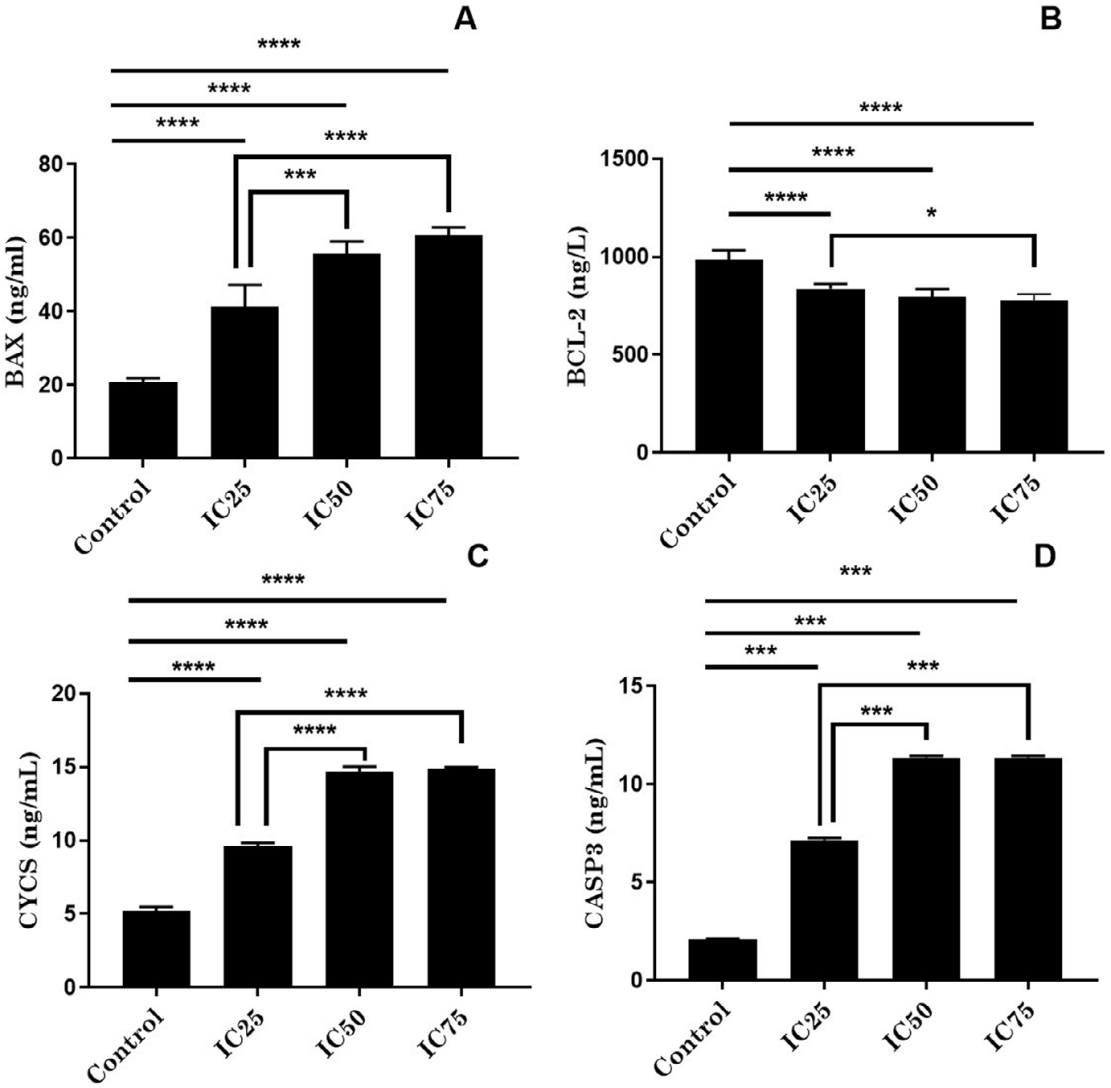
Apoptosis panel of U251 cells with BA treatment. *p < 0.05, ***p < 0.001, and ****p < 0.0001; IC25 15.62, IC50 31.25, and IC75 62.5 μg/mL BA, respectively. The results are presented as the mean ± SD.

BA dose‐dependent levels of some proteins, which are cell apoptosis markers, were determined. BAX levels, the initiator of the apoptotic process, were found to be significantly increased in BA‐treated cells compared to control cells (*p* < 0.0001, Figure 4A). There was a statistically significant increase in IC50 and IC75 treated cells compared to IC25 dose (*p* < 0.001 and *p* < 0.0001 respectively, Figure 4A). The opposite of BAX results was observed in Bcl‐2 levels, which is an anti‐apoptotic protein. Bcl‐2 levels were highly decreased in BA‐treated cells compared to control cells (*p* < 0.0001, Figure 4B), while the decrease was more dramatic at IC75 dose compared to IC25 dose (*p* < 0.05, Figure 4B). CYCS levels increased with BA treatment compared to control cells (*p* < 0.0001, Figure 4C). The increase in IC50 and IC75 doses compared to IC25 dose is statistically significant (*p* < 0.0001, Figure 4C). Finally, while CASP3 levels increased with BA application, the increase in IC50 and IC75 levels was more dramatic compared to IC25 dose (*p* < 0.001, Figure 4D).

**FIGURE 5:**
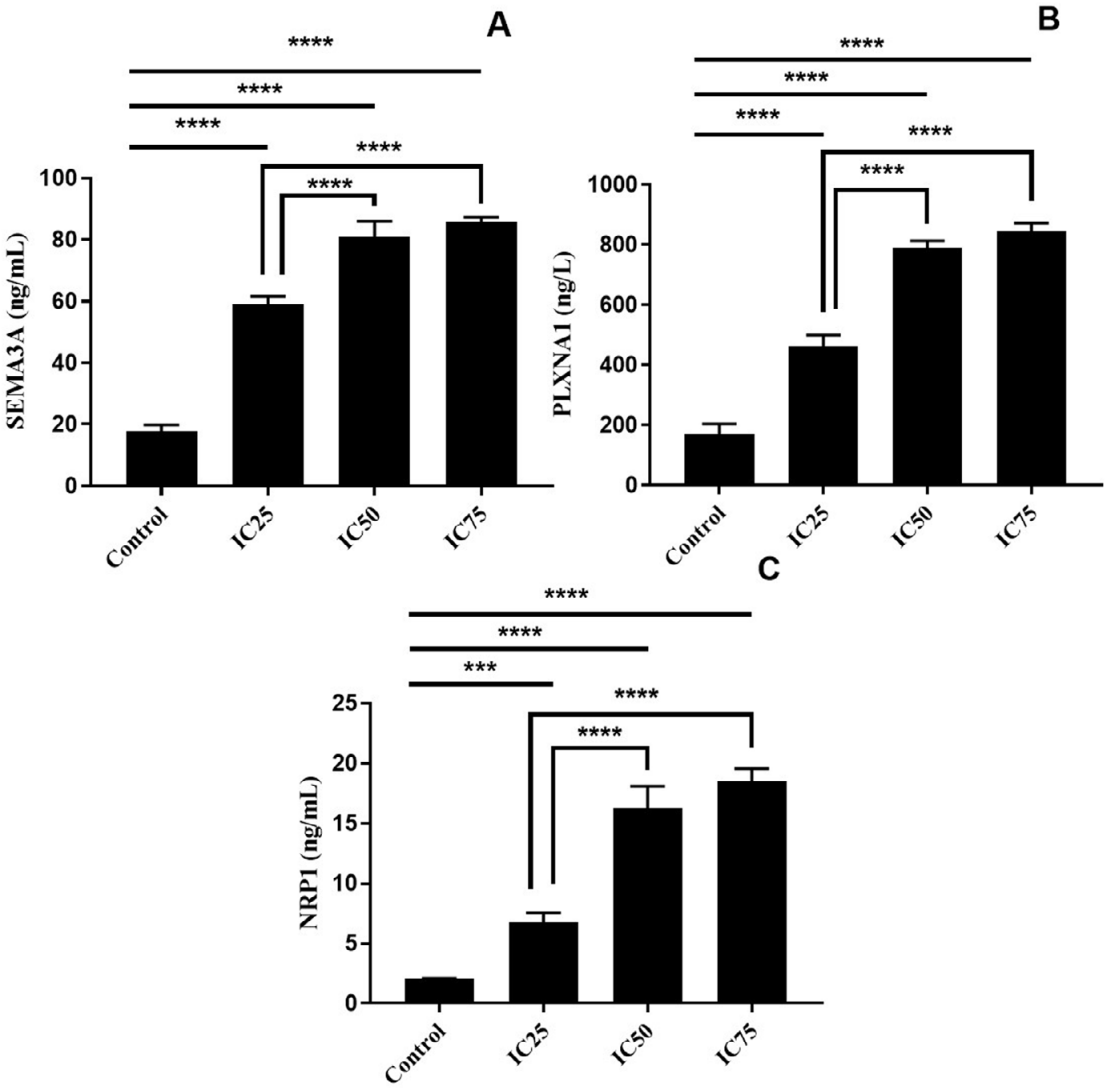
Semaphorin signalling pathway panel of U251 cells with BA treatment. A: Semaphorin‐3A (SEMA3A), B: Plexin‐A1 (PLXNA1) and C: Neuropilin‐1 (NRP1). ***p < 0.001 and ****p < 0.0001, IC25 15.62, IC50 31.25, and IC75 62.5 μg/mL BA, respectively. The results are presented as the mean ± SD.

The levels of SEMA3A and its receptor proteins were analysed after BA treatment. SEMA3A levels were significantly lower in control cells than in BA‐treated cells (*p* < 0.0001, Figure 5A). The increase in SEMA3A levels with increasing BA dose showed statistical significance. SEMA3A levels in cells treated with IC50 and IC75 doses were significantly increased compared to cells treated with the IC25 dose (*p* < 0.0001, Figure 5A). The levels of receptor proteins PLXNA1 and NRP1 increased in BA‐treated cells compared to control cells (*p* < 0.0001, Figure 5B,C), and the increase was significantly higher at IC50 and IC75 doses compared to the IC25 dose (*p* < 0.0001, Figure 5B,C).

### 3.3 Molecular Analyses Results

**FIGURE 6:**
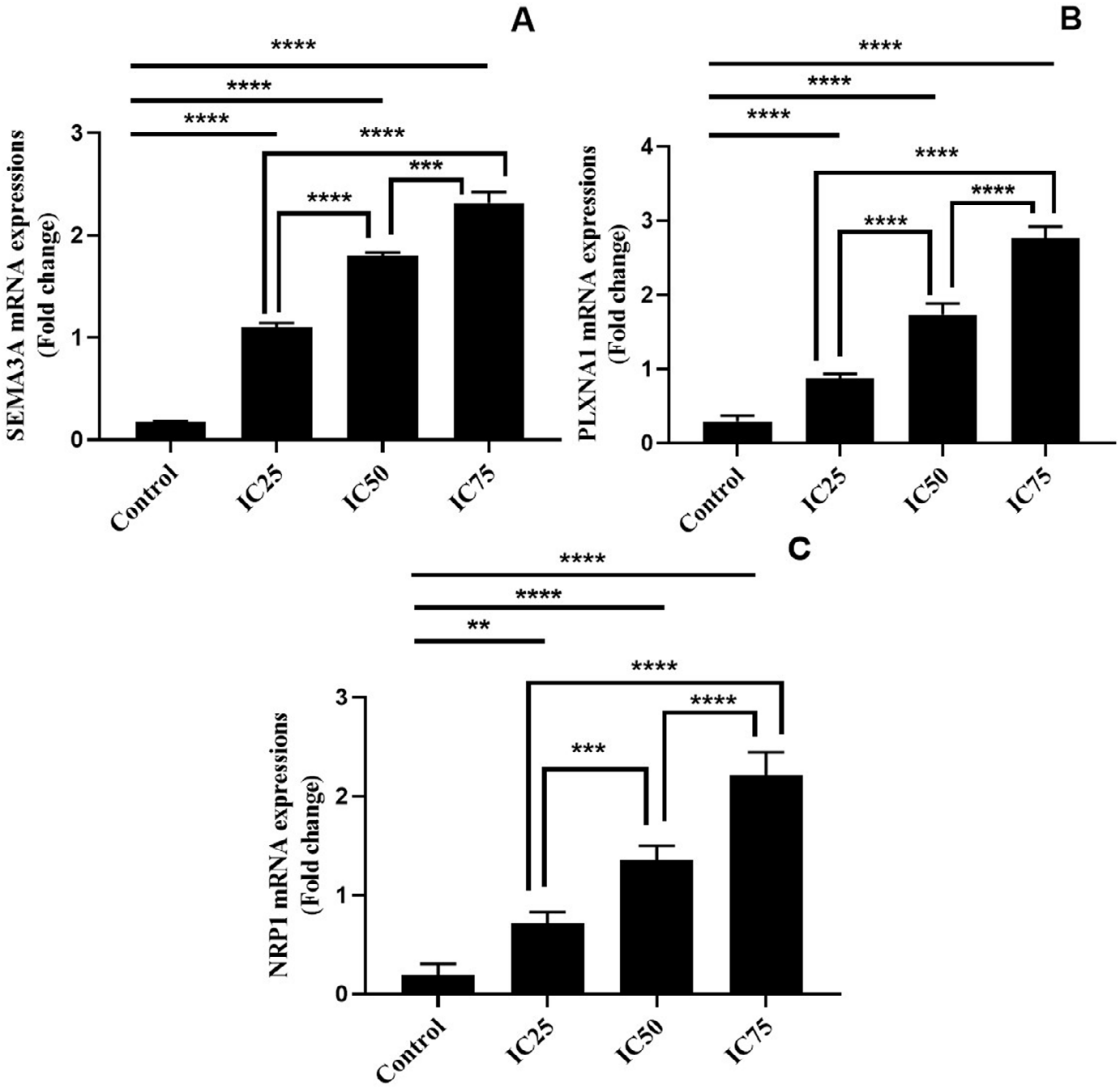
Semaphorin signalling pathway gene expression levels of U251 cells with BA treatment. A: Semaphorin‐3A (SEMA3A), B: Plexin‐A1 (PLXNA1) and C: Neuropilin‐1 (NRP1). **p < 0.01, ***p < 0.001 and ****p < 0.0001, IC25 15.62, IC50 31.25 and IC75 62.5 μg/mL BA, respectively. The results are presented as the mean ± SD.

Gene analyses were also performed to evaluate the semaphorin pathway in detail. Gene analysis results were similar to biochemical analyses. However, it was also found that there was a difference between IC50 and IC75 doses in gene analyses results. SEMA3A gene expression levels increased in BA‐treated cells compared to control cells (*p* < 0.0001, Figure 6A). At the same time, as the dose increased, SEMA3A gene expressions were found to increase significantly in a dose‐dependent manner (*p* < 0.0001, Figure 6A). The gene expression levels of receptor proteins PLXNA1 (*p* < 0.0001, Figure 6B) and NRP1 (*p* < 0.0001, Figure 6C) were found to be significantly increased in a dose‐dependent manner as in SEMA3A.

## 4 Discussion

In this study, the anti‐cancer activity of BA on human glioblastoma cells was examined through the semaphorin/plexin/neurophilin pathway. Elucidating the role of BA in cancer progression and its anti‐cancer properties is crucial, given that inorganic borate compounds exist in the human body in the form of BA [33].

BA is among the most extensively researched boron‐containing compounds and has been demonstrated to regulate the proliferation of certain cancer cell types [28, 29, 30]. In a study with the DU145 human prostate cancer cell line, it was shown that high doses of BA (12 mM) dramatically reduced cell viability in 24 h [34]. High doses of BA (30 mM) applied to hepatocellular carcinoma cells were also found to highly inhibit cell viability [27]. High dose BA treatment (12.5–50 mM) also inhibited cell growth in melanoma and MDA231 breast cancer cells [35, 36]. In our study, 15.62, 31.25 and 62.5 μg/mL BA doses were determined as IC25, IC50 and IC75 doses for U251 cells, and incubation time was chosen as 24 h. Notably, while previous studies have required substantially higher concentrations of BA to achieve cytotoxic effects in various cancer cell lines, our study demonstrates that boric acid is effective at much lower doses in U251 glioblastoma cells. This highlights the heightened sensitivity of U251 cells to boric acid and suggests its potential as a therapeutic agent at lower, potentially less toxic concentrations. BA shows inhibitory effects on enzymes such as peptidase, proteinase and arginase [37]. The reduction of L‐arginine through the inhibition of myeloid cell arginase hinders the targeted immune functions of T cells, constituting a primary method by which cancer circumvents immunosurveillance [38]. Due to their ability to form complexes with serine hydroxyl groups, BA and its products inhibit serine proteases such as thrombin and specific coagulation factors. Serine proteases are involved in the aggregation of tumour cells [39]. The aggregation of tumour cells and their clumping with platelets can shield circulating tumour cells from shear stress and immune attacks [40]. Therefore, the inhibitory effects of BA products on serine proteases and their anticoagulant properties may potentially reduce metastasis.

Oxygen is indispensable for life, yet its consumption has costs that can disrupt the delicate balance necessary for survival. ROS are oxygen derivatives characterised by one or more unpaired electrons in their outermost shell, originating from molecular oxygen [41]. When antioxidant agents fail to detoxify ROS, oxidative stress ensues, causing damage to biomolecules such as proteins and DNA. This can lead to severe cellular damage, including chromosomal aberrations, epigenetic dysregulation and deterioration of the cell cycle [42]. Interestingly, the generation of ROS is a widely utilised mechanism in many anticancer treatments, such as chemotherapy, radiotherapy and photodynamic therapy, due to their role in inducing cell death [43]. In the study conducted with U87MG glioblastoma cells, it was found that apoptosis was induced by increasing ROS production, and cell death occurred in cells treated with Curcumin and Temozolomide [44]. Evidence has been presented that ROS levels are greatly increased after luteolin treatment of U87MG and U251MG cells and support the mechanism of programmed cell death [45]. In the present study, TAS, TOS, OSI, and GSH levels were measured to examine the oxidative balance in U215 cells after BA treatment. After increasing BA doses, TAS and GSH levels decreased, whereas TOS and OSI levels increased. This situation reveals the findings that BA promotes cell death by triggering ROS formation in cancer cells.

Apoptosis is a process of programmed cell death that controls cell growth. The primary mechanism of commonly used anticancer drugs such as tamoxifen is to suppress cell growth and induce apoptosis in cancer cells [46]. Two significant members of the Bcl‐2 protein family, Bcl‐2 and BAX, are essential for controlling apoptosis. While BAX is a pro‐apoptotic protein that encourages apoptosis, Bcl‐2 is an anti‐apoptotic protein that suppresses it [47]. ROS frequently triggers the apoptotic pathways by increasing the permeability of the mitochondrial membrane. This leads to the release of pro‐apoptotic molecules into the cytosol, including CYCS, an intermembrane space protein that is in charge of mitochondrial electron transport [48]. Glioma cells have decreased activity of CASP3, a crucial mediator of the apoptosis pathway, which increases the cells' resistance to apoptosis and encourages the formation of tumours [49]. It is known that CASP3 activity and apoptosis in glioma cells are regulated by the balance between pro‐apoptotic BAX and anti‐apoptotic Bcl‐2 proteins [50]. The anti‐cancer activity of Tubeimoside‐1 in human glioma cell lines was investigated through apoptotic processes. It was found that apoptosis was induced in cancer cells by increasing BAX levels and decreasing Bcl‐2 levels; CYCS and CASP3 levels, which are ROS‐assisted apoptotic markers, also increased [51]. In the anticancer efficacy study of medicarpin on U251 and U87 glioblastoma cells, it was found that medicarpin increased apoptosis in cancer cells by increasing BAX, CYCS and CASP3 gene levels and also decreased Bcl‐2 levels [52]. We found that BAX, CYCS and CASP3 levels amplified and Bcl‐2 levels reduced in U251 glioblastoma cells to which we applied BA at different doses. Dose‐dependently, BA, especially with the effect of increased ROS, may have activated the intrinsic pathway of apoptosis [53], induced BAX levels, increased CYCS levels by disrupting mitochondrial membrane permeability, and activated CASP3 protein.

In vitro and in vivo research have demonstrated the growing body of evidence supporting the participation of specific semaphorins as important regulators of cancer cell growth and survival [6, 54, 55]. Remarkably, it was stated that overexpressing secreted SEMA3A and Sema3F significantly decreased colony formation and proliferation in soft agar in human GBM cells U87MG, while the expression of homologous family members SEMA3B, SEMA3D, and SEMA3G did not significantly alter the culture [56]. In a recent study with gastric cancer cells, SEMA3A overexpression was shown to arrest the proliferation, migration, and metastatic properties of cancer cells [57]. Both in vivo and in vitro results in a xenograft oral cancer model showed that SEMA3A overexpression halted tumour cell growth by inhibiting angiogenesis [58]. In the present study, SEMA3A, its receptor PLXNA1, and its co‐receptor NRP1 levels were analysed by BA treatment in U251 cells. It was determined that the production of SEMA3A and its receptors was induced as the dose of BA application increased. Also, in gene expression levels, a statistically significant increase was found between all BA doses. These results reveal that BA administration in a dose‐dependent manner can arrest tumour growth by inducing overexpression of SEMA3A and its receptors.

Our study demonstrated the efficacy of BA on cancer cells through different mechanisms. There are different opinions in the literature on the necessity and safety of clinical use of BA. BA is considered minimally to mildly toxic in small doses, with limited dermal and respiratory effects in humans. However, high or chronic exposure—especially in infants or through ingestion—can lead to serious health issues such as skin irritation, gastrointestinal symptoms, seizures and reproductive toxicity. While it shows no carcinogenic or mutagenic potential, animal studies indicate testicular and developmental toxicity at high doses, though similar effects are not consistently seen in human studies [59]. Therefore, it is extremely important to increase in vitro and in vivo analyses for BA, which clearly has anti‐cancer activity, before proceeding to clinical trials.

In this study, the effect of a specific dose of BA on U251 cells was evaluated. Our hypothesis and results provided evidence that BA inhibits tumour progression by regulating apoptosis and the semaphorin signalling pathway. To the best of our knowledge, BA levels and the effect of the semaphorin signalling pathway were evaluated for the first time with U251 cells. We showed that BA inhibits the growth of tumour cells by inducing the SEMA3A/PLXNA1/NRP1 pathway and prevents cell growth by inducing oxidative damage and activating apoptosis through excessive ROS generation. The availability of BA‐induced oxidative stress and SEMA3A biomarkers in a dose‐dependent manner, the mechanism of apoptosis, and the effective dose of BA can be evaluated for the focus of future pre‐clinical studies on glioblastoma.

## Author Contributions

**Ezgi Kar:** conceptualization (equal), investigation (equal), visualization (equal), writing – original draft (lead). **Zeynep Övenler:** investigation (equal), methodology (equal), writing – review and editing (equal). **Ceyhan Hacıoğlu:** formal analysis (equal), investigation (equal), resources (equal), writing – review and editing (equal). **Fatih Kar:** conceptualization (equal), investigation (equal), validation (equal), writing – review and editing (equal).

## Ethics Statement

The authors have nothing to report.

## Consent

The authors have nothing to report.

## Conflicts of Interest

The authors declare no conflicts of interest.

## Data Availability

The data that support the findings of this study are available from the corresponding author upon reasonable request.
